# Assessment of oral manifestations in pediatric patients with celiac disease in relation to marsh types

**DOI:** 10.4317/medoral.25490

**Published:** 2022-12-24

**Authors:** Cigdem Elbek-Cubukcu, Hanife Aysegul Arsoy, Guven Ozkaya

**Affiliations:** 1 Assoc. Prof., Bursa Uludag University, Faculty of Dentistry, Department of Pedodontics, Bursa, Türkiye; 2 MD., Bursa Uludag University, Faculty of Medicine, Department of Pediatric Gastroenterology, Hepatology and Nutrition, Bursa, Türkiye; 3 Prof., Bursa Uludag University, Faculty of Medicine, Department of Biostatistics, Bursa, Türkiye

## Abstract

**Background:**

To investigate the presence of molar-incisor hypoplasia and recurrent aphthous ulcers, the level of caries experience, and oral hygiene status, and to measure salivary flow rate, salivary buffer capacity, and salivary cariogenic microflora with Marsh types.

**Material and Methods:**

A single-blind, prospective clinical study with 62 pediatric patients diagnosed with celiac disease with 64 controls. Clinical identification of molar-incisor hypomineralization (MIH) was followed according to the European Academy of Pediatric Dentistry criteria. DMFS and dfs index were used for the caries experience of each child. The clinical diagnosis of RAU was present or not. Oral hygiene was surveyed by recording the OHI-S and the CRT® Bacteria and Buffer Test was used to examine the cariogenic microflora of each child.

**Results:**

The prevalence of MIH was 61% and the number of recurrent aphthous ulcers were significantly higher in children with celiac disease. There was no statistically significant difference in the CD group, when DMFS, dfs, and MIH parameters were investigated according to dietary compliance. Higher dietary compliance resulted in better oral hygiene status. There was an inverse relationship between the duration of celiac diagnosis and the presence of MIH. A positive relation was found between the duration of the disease and the severity of MIH. In addition to the higher *S. mutans* counts, the salivary flow rate was very low in children with celiac disease, indicating a positive correlation between poor dietary compliance and poorer oral hygiene.

**Conclusions:**

In children, enamel defects and recurrent mucosal lesions may be a sign of celiac disease. Higher numbers of dental caries in permanent teeth of children with celiac disease may be related to Marsh 2 type. The pediatricians and/or pediatric gastroenterologists should refer the chin with celiac disease to the pediatric dentist for the accurate treatment of intraoral manifestations of the disease itself.

** Key words:**Celiac disease, molar incisor hypomineralization, oral ulcer, dental caries, saliva.

## Introduction

Celiac disease (CD) has been defined as an immune-mediated systemic disorder caused by prolamins (gluten) in genetically predisposed individuals (1). Its prevalence in children is 1%-8,3% and the sex distribution is similar (2,3,4). In addition to systemic symptoms of the disease, some oral manifestations such as Molar Incisor Hypomineralization (MIH), Recurrent Aphthous Ulcers (RAU), poor oral hygiene, and dental status may occur (5). MIH is a type of enamel defect which affects permanent first molars and, to a lesser degree, permanent incisors with variable severity and prevalence (2.8%-25%) (6). Clinically, demarcated opacities of white, yellow, or brown coloration may facilitate the development of dental caries (7). Microbiological examinations (salivary *S. mutans* and *Lactobacilli* counts, salivary buffering capacity, and salivary flow) are important diagnostic tools in preventing dental caries and improving the dental and oral health of children with CD (8). RAU is the most common oral cavity inflammatory condition characterized by multiple recurrent small, round or ovoid ulcers (9,10). The objectives of this study were to determine the prevalence of MIH and RAU, dental caries, and oral hygiene experiences and to measure salivary flow rate, salivary buffer capacity, and salivary cariogenic microflora in CD and healthy children. Any correlation between the CD group's oral manifestations and mucosal damage degree was also investigated.

## Material and Methods

- Study design

This study was approved by the Ethics Committee of Bursa Uludag University (process n. 2011-KAEK-26/19). This study was performed in accordance with the ethical rules of the Declaration of Helsinki, including all amendments and revisions. Informed written consent about the study was obtained.

In the Pediatric Gastroenterology outpatient clinic of the Health Sciences University Bursa Yüksek İhtisas Training and Research Hospital, 104 children previously diagnosed through a positive anti-endomysial-antibody test (IgA) and a definitive confirmation through a small-bowel biopsy associated with positive serology for CD according to the Marsh-Oberhuber classification (Marsh 1: Normal mucosa, celiac disease unlikely; Marsh 2: Hyperplastic lesion may indicate celiac disease; Marsh 3: Destructive lesion, a spectrum of changes characteristic of untreated celiac disease. Patients may be symptomatic or asymptomatic.) The study was referred to the Pedodontics Department of Dental Faculty of Bursa Uludag University between January and August 2021. Their prenatal, perinatal, and postnatal events and past CD history such as intercurrent events during pregnancy, such as the use of medications, high-grade maternal fever, and episodes of infection, comorbidities might have occurred in childhood, such as mumps, chickenpox, nutritional deficiencies, otitis, urinary tract infections were investigated. The type of CD (the classical, non-gastrointestinal and asymptomatic) and age at diagnosis were also recorded.

The exclusion criteria were children who presented with dental fluorosis, used fixed orthodontics, and dental enamel defects associated with other systemic diseases/conditions than CD such as respiratory disorders, infectious diseases in young childhood, Type 1 Diabetes, syndromes (Down S., Turner S., etc.) and premature birth. Children who used any type of medicine in the moment of the clinical analyses were also excluded. Forty-two of them were excluded from the study group according to the exclusion criteria. The study group consisted of 62 children with CD.

Eligible 64 healthy children with demographic characteristics similar to the study group who attended the Pedodontics Department for their regular dental appointments or first visit were recruited as a control group.

**Figure 1:**
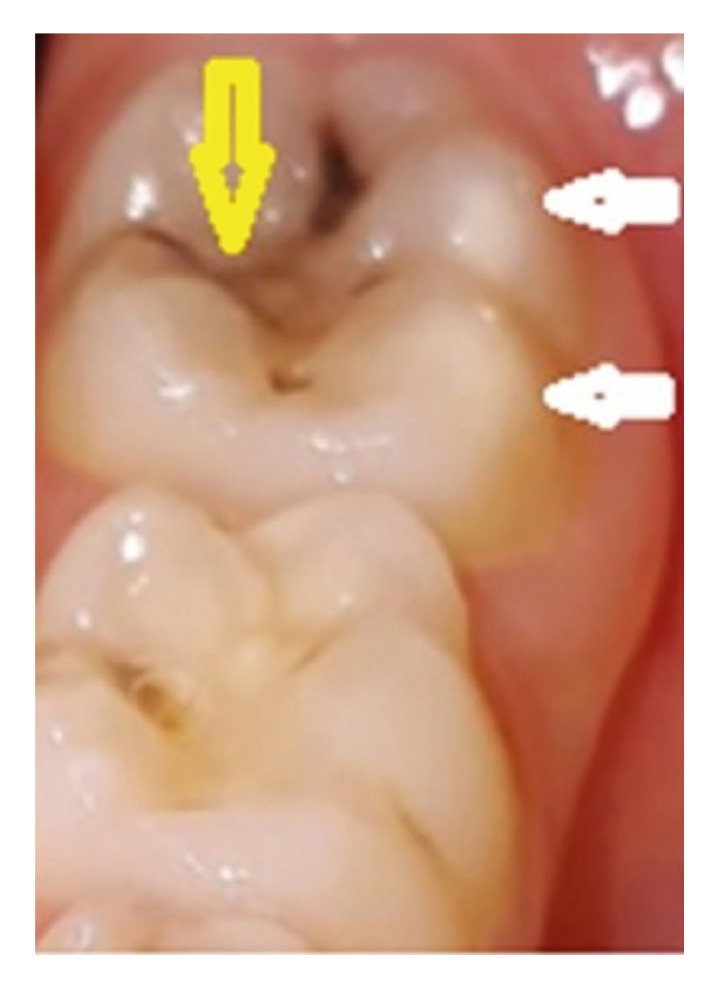
Permanent lower left first molar of a 7-year-old boy in the CD group. A yellow arrow indicated caries on the occlusal surface and two white arrows showed MIH on buccal tubercules.

The oral examination was carried out in a dental chair with a mouth mirror and blunt dental probe under artificial light in the Pedodontics Clinic. Clinical identification of MIH was followed according to the European Academy of Pediatric Dentistry (EAPD) criteria in which at least one first molar must have demarcated opacity, the presence of atypical restorations, post-eruptive fracture, or extraction due to condition (Fig. 1).

**Figure 2:**
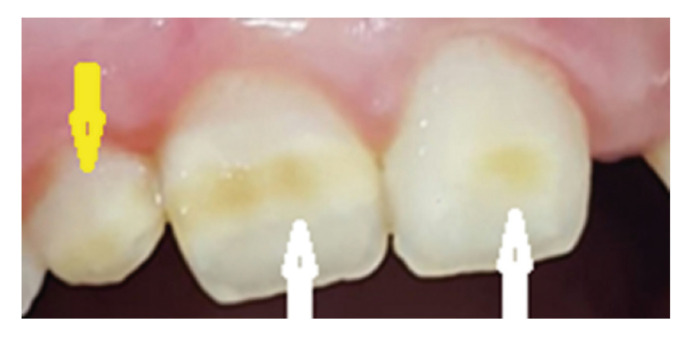
The MIH on the permanent upper central incisors of a 9 years old girl in the CD group (white arrows). An enamel defect was also indicated on the right upper lateral incisor (yellow arrow) however the tooth was not within the scope of MIH investigation.

Permanent central incisors were also evaluated (Fig. 2).

The demarcated opacities were considered mild injuries, while atypical restorations, post-eruptive fracture, or MIH extraction were considered severe. Only defects greater than 1.0 mm in diameter were evaluated (11). Relations between oral manifestation and intestinal histopathological changes according to the Marsh-Oberhuber classification were also analyzed.

**Figure 3:**
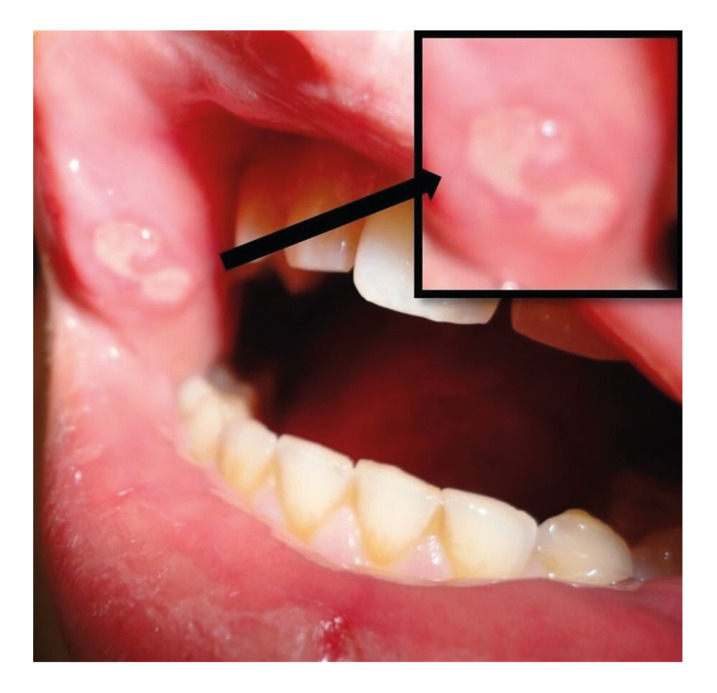
RAUs on the right buccal mucosa of a 14-year-old boy with poor oral hygiene in the CD group. A black arrow indicated the aphthous lesion.

To determine the caries experience of each child, DMFS (the sum of the number of Decayed, Missing due to caries, and Filled tooth Surfaces in the permanent teeth) and dfs (the sum of the number of decayed, and filled tooth surfaces in the primary teeth) index were used by a previously calibrated pediatric dentist (12). The same examiner examined the soft tissues for the diagnosis of RAU as shown in Fig. 3 (present or not) and the patient’s parents were also questioned about the recurrent occurrence of oral ulcers.

Oral hygiene was surveyed by recording the Simplified Oral Hygiene Index (OHI-S) consisting of two components that are based on numerical determinations representing the amount of debris or calculus found on the pre-selected tooth surfaces (12).

Following initial oral examination was completed another dental appointment was scheduled. The microbiological tests were applied to those who had not used antibiotics and/or antimicrobial solutions in the last 7 days before the saliva sample collection. The CRT® Bacteria and Buffer Test (Ivoclar Vivadent, Liechtenstein) was used to examine the cariogenic microflora of each child. The salivary buffering capacity was determined with a colorimetric test strip. Patients were asked not to eat or drink anything 90 minutes before saliva collection. Children were placed in a relaxed position to looking downwards and stimulated to salivate by chewing paraffin pieces for 5 minutes, and saliva was collected in a calibrated container. Saliva buffering capacity and salivary secretion rate (ml saliva/min) were calculated simultaneously by the manufacturer's recommendations. The collected saliva to determine its buffering capacity was also used for the detection of bacteria: the density of *S. mutans* and *Lactobacilli* in the saliva was measured by selective agar cultures. The agar carrier was removed from the test tube. A sodium bicarbonate Tablet was placed at the bottom of the tube. Excess saliva was allowed to drain from both agar surfaces. The removed agar carrier was placed back in the tube and the tube was tightly capped. Culture tubes were incubated at 37ºC for 48 hours. The density of *S. mutans* and *Lactobacilli* colonies was evaluated according to the scale accompanying the test.

- Sample size

The sample size was determined based on power analysis calculations. Power analysis was performed using G power ver. 3.1.9.7 (http://www.gpower.hhu.de). The primary endpoint was the MIH variable. The effect size between CD children and control was accepted as 0.50 (medium effect size) and was used for determining the total sample size, using a power of 80% and an alpha level of 5%. The minimum required sample size was calculated as 62 children for each group.

- Statistical analysis

Statistical analyses were performed with IBM SPSS ver.23.0. Descriptive statistics were given mean ± standard deviation, median (interquartile range), or frequency with percentage. Differences between CD children and the control group were tested using the chi-square test or Fisher’s exact test for categorical variables and the Student t test or Mann–Whitney test for continuous variables. Kruskal Wallis test was used for continuous variables between Marsh types. A *P* value of less than 0.05 was considered statistically significant.

## Results

The distribution of sex was homogenous both within and between the groups. The mean age of CD diagnosis was 9.17±4.39. The classical type of CD had the largest percentage (n=39, 31%) in the study group. The non-gastrointestinal and asymptomatic CD numbers were 10 (7.9%) and 13 (10.3%), respectively. More than half of the subjects with CD (n=33, 53.2%) followed a gluten-free diet.

Descriptive statistics regarding the sociodemographic characteristics and oral clinical findings of the study population were given in Table 1. A statistically significant difference was found in the mean number of RAU and MIH which were higher in children with CD compared to the control group (Table 1, *P*<0.001).

MIH prevalence was found at 61% in children with CD. The total percentage of children without MIH was 52.0% (n=65). While the percentage of MIH was 37.7% (n=23) for the CD group, it was found 65.6% (n=42) for the controls. The percentage of children with MIH>4 (including permanent incisors) was 11.7% in the CD group. In the control group, there were no children with MIH>4. In CD children, a moderately significant inverse relationship was found between the number of MIH and age at diagnosis (r= -0.605, *P*<0.001) However, a positive relation was found in the duration of the CD for the number of MIH (r=0.536, *P*=0.001).

There was no statistically significant difference in the CD group, when DMFS, dfs, and MIH parameters were investigated according to dietary compliance (gluten-free diet). However, it was not true in terms of the OHI-S variable which was found lower in children stuck with a gluten-free diet than in those who did not (Table 2, *P*=0.011).

Results as percentages of cariogenic flora counts and saliva tests in CD children compared to controls were presented in Table 3. In the CD group, very low saliva buffering capacity (<0.7 ml/min) and saliva flow was determined significantly (*P*<0.001) compared to controls. Additionally, >105 *S. mutans* counts (72.6%) were found significantly higher than the controls (27.4%) (*P*=0.003). When it was compared the *S. mutans* counts and salivary parameters in the study, there was a positive correlation was determined between the gluten-free diet poor compliance (r=0.567) and OHI-S (r=0.358) which were insignificant.

**Figure 4:**
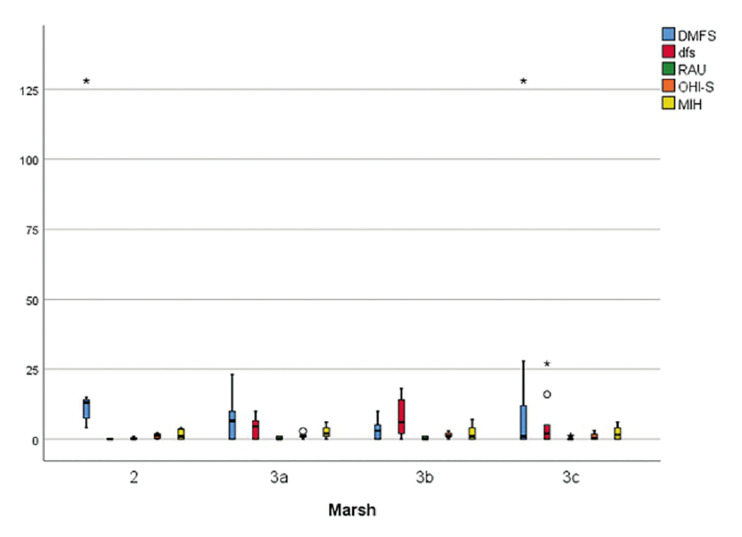
Box plot graph of MIH, RAU, DMFS, dfs, and OHI-S variables according to Marsh types.

While there was a statistically significant difference for the DMFS (*P*=0.038) according to Marsh types among the children with celiac disease, no significant difference was found for dfs, RAU, OHI-S, and MIH variables (Table 4). As a result of pairwise comparisons, the DMFS value of the Marsh 2 group was found to be higher than Marsh 3b and 3c (Fig. 4).

**Table 1:**
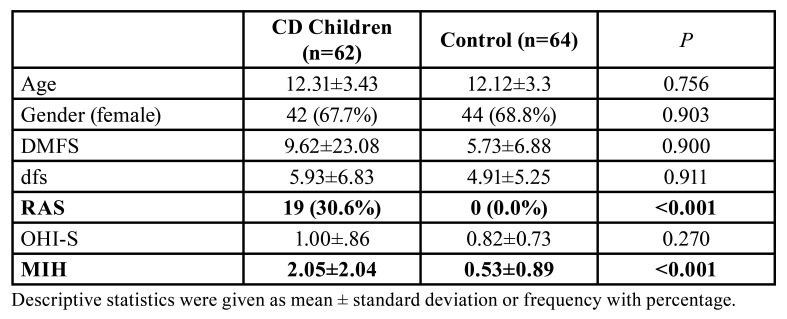
Descriptive statistics regarding the sociodemographic characteristics and oral clinical findings of both CD children and controls.

**Table 2:**
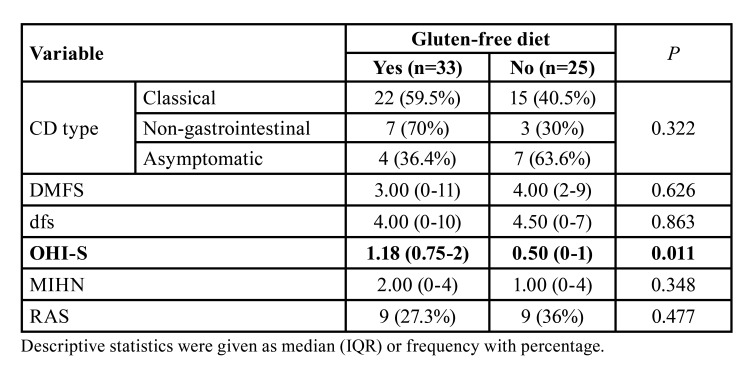
Comparison of variables according to dietary compliance (gluten-free diet) of CD children.

**Table 3:**
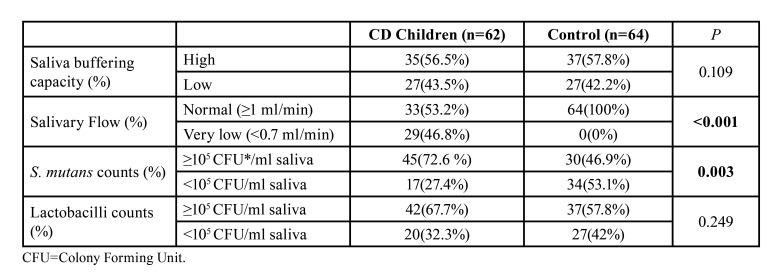
Cariogenic flora and saliva tests in CD children compared to controls.

**Table 4:**
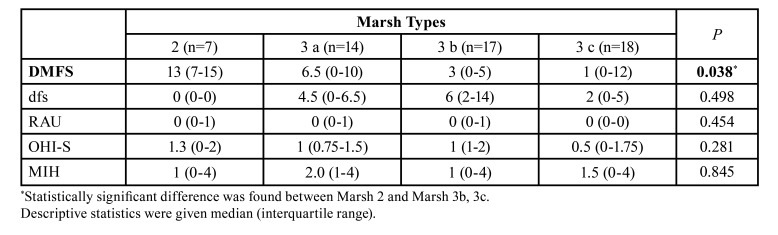
Comparison of variables according to Marsh types in children with celiac disease.

## Discussion

The critical period between the 28th week in utero life to the first 10 days of life after birth, is very important because the maturation phase of amelogenesis (tooth enamel formation) of the first permanent molars and permanent incisors begins (13). Any risk factors such as CD during the maturation phase in which enamel will mineralize cause teeth hypomineralization (14). The resulting enamel is not completely mineralized, causing yellowish, hypersensitive enamel, with little resistance affecting the dentin’s surrounding tissue as in MIH (7). The North American Society for Pediatric Gastroenterology, Hepatology, and Nutrition (NASPGHAN) included the presence of MIH as a risk factor for CD which could be a major sign of the disease (15). Studies reported significantly higher in CD patients than healthy children (6,16-21). Kuklik *et al*. reported children with CD presented 4.75 times the chance of occurrence of MIH than the controls and all CD subjects with MIH presented the classic form of the CD which was in our study (22). Avşar and Kalaycı, Acar *et al*., Cantekin *et al*. and Dane and Gürbüz reported a higher prevalence of enamel defects in children with CD than in controls which were 42.2%, 40%, 48%, and 54.3%, respectively (16-19).

RAU is the most common oral cavity inflammatory ulcerative condition and is characterized by multiple recurrent small, round, or ovoid ulcers with circumscribed margins, erythematous haloes, and yellow or gray floors. It is more prevalent in children with nutritional- and immuno- deficiencies, malabsorption, and CD (9,10). In our study, RAU prevalence in the CD group was significantly higher (30.6%, *P*<0.001) compared to controls. In previous studies prevalences of RAU in children with CD were 22.7%, 26%, and 8.3%, respectively which were lower compared to our results for RAU (21-23). However, the prevalence of RAU was previously reported as 33.3% in CD children by Bucci *et al*. similarly to our study (24).

There was no statistical significance was found between the types of CD and the presence of RAU in the study. Some reports are indicating there is a relationship between them (24-27). In the study of Bramanti *et al*., RAU was found in 52% of ascertained celiac patients and 66.7% of potential celiac patients (25). However, in our study, no potential type of CD children was included. Macho *et al*. also reported that RAU was more frequent in silent (asymptomatic) celiac patients, who did not report any gastrointestinal symptoms before the diagnosis of CD (26). Therefore, RAU could be considered a sign of the atypical or silent forms of CD.

Concerning the relationship between dental caries and CD, some studies reported a lower dental caries prevalence than another reported higher (6,17,21,27). Avşar and Kalaycı found that caries-free subjects in matched controls were higher (38%) compared to CD children (17%) (16). Cantekin *et al*. reported that DMFT values of CD children were significantly different from the controls (3.75 vs. 1.83) (18). However, Acar *et al*. reported similar DMFS and dfs values for CD children and their matched controls which were similar to our study (17). The controversial results could be related to gluten-free diet compliance, the degree of severity and the higher number of hypoplastic enamel (MIH), deficiencies in salivary composition, and flow rate as the risk factors for dental caries (21). However, in our study, DMFS and dfs values were not associated with compliance to a gluten-free diet in both children with CD and controls. Dental caries experiences were not found to be lower in those who had compliance with the gluten-free diet than in those who did not.

OHI-S grades indicated insignificance between the groups in our study. However, OHI-S values were found significantly lower in children with CD who had been following a gluten-free diet. In a study from Israel OHI-S grades were significantly correlated with the presence of a coexisting CD and frequency of tooth brushing (28). However, in another study results indicated a lower degree of bacterial plaque was found on the teeth of children with CD receiving a gluten-free diet and performing better oral hygiene (29). Therefore, all children included in the study should be educated about oral health and the importance of gluten-free (anti-cariogenic) nutrition.

In this study, the salivary flow rate was lower in children with CD compared to controls. Counts of salivary *mutans streptococci* were found to be significantly higher in CD patients however it was not true for the salivary *Lactobacilli* counts. Acar *et al*. reported that salivary parameters were similar in both CD and control groups whereas salivary *mutans streptococci* and *lactobacilli* counts were found to be significantly lower in CD patients compared to healthy subjects (17). Although no previous scientific literature other than the study mentioned above could be provided, it could be expected that the anti-cariogenic (gluten-free) diet might inhibit the cariogenic microflora and conclude that good gluten-free dietary compliance was very important.

Our results indicated a significant correlation between the DMFT variable and Marsh types among the children with CD. Children who had Marsh 2 scores had higher numbers of dental caries in permanent dentition. No other scientific literature was determined to give any information about the relationship between the histopathological changes of the disease and the presence of oral manifestations and this was the first study that investigated the relationship between oral manifestations and Marsh types of celiac disease.

This study analyzed specific oral manifestations in children with celiac disease in comparison with non-celiac controls. The preventive recognition of specific dental and oral mucosal lesions especially by pediatricians and dentists is of great importance. Examination of the oral cavity with particular attention to dental enamel defects and mucosal ulcerations may contribute to an early diagnosis of celiac disease and may prevent its progress and complications.

- Limitations of the study

It is recommended that the study sample be larger in number so that the intraoral manifestations of celiac disease in children can be categorized according to Marsh types.
